# Characterization of the ER-Targeted Low Affinity Ca^2+^ Probe D4ER

**DOI:** 10.3390/s16091419

**Published:** 2016-09-02

**Authors:** Elisa Greotti, Andrea Wong, Tullio Pozzan, Diana Pendin, Paola Pizzo

**Affiliations:** 1 Department of Biomedical Sciences, University of Padua, Via U. Bassi 58/B, Padua 35121, Italy; e.greotti@gmail.com (E.G.); andreakuancie.wong@gmail.com (A.W.); tullio.pozzan@unipd.it (T.P.); paola.pizzo@unipd.it (P.P.); 2 Neuroscience Institute—Italian National Research Council (CNR), Padua 35121, Italy; 3 Venetian Institute of Molecular Medicine, Padua 35121, Italy

**Keywords:** Calcium, Endoplasmic reticulum, Cameleon, FRET-based probe, Presenilin

## Abstract

Calcium ion (Ca^2+^) is a ubiquitous intracellular messenger and changes in its concentration impact on nearly every aspect of cell life. Endoplasmic reticulum (ER) represents the major intracellular Ca^2+^ store and the free Ca^2+^ concentration ([Ca^2+^]) within its lumen ([Ca^2+^]_ER_) can reach levels higher than 1 mM. Several genetically-encoded ER-targeted Ca^2+^ sensors have been developed over the last years. However, most of them are non-ratiometric and, thus, their signal is difficult to calibrate in live cells and is affected by shifts in the focal plane and artifactual movements of the sample. On the other hand, existing ratiometric Ca^2+^ probes are plagued by different drawbacks, such as a double dissociation constant (K_d_) for Ca^2+^, low dynamic range, and an affinity for the cation that is too high for the levels of [Ca^2+^] in the ER lumen. Here, we report the characterization of a recently generated ER-targeted, Förster resonance energy transfer (FRET)-based, Cameleon probe, named D4ER, characterized by suitable Ca^2+^ affinity and dynamic range for monitoring [Ca^2+^] variations within the ER. As an example, resting [Ca^2+^]_ER_ have been evaluated in a known paradigm of altered ER Ca^2+^ homeostasis, i.e., in cells expressing a mutated form of the familial Alzheimer’s Disease-linked protein Presenilin 2 (PS2). The lower Ca^2+^ affinity of the D4ER probe, compared to that of the previously generated D1ER, allowed the detection of a conspicuous, more clear-cut, reduction in ER Ca^2+^ content in cells expressing mutated PS2, compared to controls.

## 1. Introduction

Cell survival relies on complex signal pathways shaped by dynamic changes, both in time and space, of second messengers’ concentration. Among them, Ca^2+^ is one of the most important. In resting conditions, cells are able to maintain a large [Ca^2+^] gradient between the cytosol ([Ca^2+^]~100 nM) and the extracellular medium ([Ca^2+^]~1.5–2 mM). Maintenance of this gradient is ensured by the activity of exchangers, pumps and buffers and by Ca^2+^ compartmentalization in organelles [1]. Changes in cytosolic [Ca^2+^], caused by its influx from the extracellular medium or release from intracellular stores, modulate a plethora of key cellular events, ranging from exocytosis to muscle contraction, adenosine triphosphate (ATP) production and transcription activation. 

In mammalian cells, the main Ca^2+^ stores are represented by the ER and the Golgi apparatus (GA), while the role of acidic compartments (granules, lysosomes, endosomes) in cellular Ca^2+^ handling is still debated since the [Ca^2+^] measured varies depending on the cell type and the experimental conditions. The luminal [Ca^2+^] of the ER is rather high (estimates vary from 200 μM to over 1 mM) [2]. Maintaining a correct [Ca^2+^] within the ER is of crucial importance for cell viability and the steady state level depends on the equilibrium between Ca^2+^ leak and uptake. In turn, Ca^2+^ accumulation depends on the activity of Sarco/Endoplasmic Reticulum Calcium ATPases (SERCAs), a family of transmembrane proteins that pump two Ca^2+^ ions into the ER lumen per molecule of hydrolysed ATP [3]; Ca^2+^ storage depends on the expression of low affinity-high capacity Ca^2+^-buffering proteins within the ER lumen, while Ca^2+^ release upon stimulation relies primarily on two Ca^2+^ channels localized on ER membranes, the so-called inositol 1,4,5-triphosphate (IP_3_) receptors (IP_3_Rs), ubiquitously expressed, and the ryanodine receptors (RyRs), mainly found in excitable cells. The activation of these channels induces a rapid efflux of Ca^2+^ from the ER lumen to the cytosol [2]. Recently, a new ER Ca^2+^ channel, named Ca^2+^ load-activated Ca^2+^ (CLAC) channel, has been described which responds to ER luminal Ca^2+^ overload by homotetramerization. CLAC channels appear to function as release channels under conditions of excessive Ca^2+^ accumulation in the ER lumen [4]. Despite its undisputed role in mammalian cells, it has to be noted, however, that, in plants, the ER seems to cover a different role in cell Ca^2+^ homeostasis, with the organelle dynamically following cytosolic Ca^2+^ rises instead to be a major intracellular store involved in stimulation-induced Ca^2+^ releases [5].

The Ca^2+^-dependent cross-talk between ER and other organelles is pivotal in cell physiology. For example, the ER-mitochondria interaction, and the Ca^2+^ exchange between the two organelles, regulates mitochondrial metabolic activity (e.g., ATP production) and any alteration in this pathway impacts on cell survival [6]. Indeed, a growing body of evidence is emerging suggesting that Ca^2+^ dysregulation and alterations of ER-mitochondria Ca^2+^ cross-talk are key events in the pathogenesis of many neurodegenerative diseases, such as Alzheimer’s or Parkinson’s Diseases (AD and PD) [7]. For example, several studies have demonstrated a link between mutations in Presenilins (PSs) and ER Ca^2+^ content alterations in familial forms of AD (FAD) (reviewed in [8,9]).

Given the importance of ER Ca^2+^ signalling in different physiological and pathological conditions, the development of tools to evaluate the dynamics of [Ca^2+^] inside the ER lumen ([Ca^2+^]_ER_) is of crucial importance. The first ER-targeted genetically-encoded Ca^2+^ indicator (GECI) was an aequorin construct specifically targeted to the ER lumen that, however, has been used exclusively for measurements in cell populations [10]. As to GECIs suitable for single cell analysis, in the last decade different single fluorescent protein (FP) Ca^2+^ sensors targeted to the ER have been generated: R-CEPIA1er and G-CEPIA1er [11], ER-LAR-GECO1 [12], CatchER [13], GCaMPer [14]. These non-ratiometric probes are, in general, very difficult to calibrate in live cells and their use is primarily qualitative.

Ratiometric probes, instead, allow [Ca^2+^] measurements independently from probe expression levels, shifts in the focal plane, and movements of the sample. Only few ratiometric probes have been targeted to the ER; one of them, named GAP3-aequorin, is a dual-excitation ratiometric probe, based on the molecular pair aequorin/GFP [15]. Furthermore, a promising class of ratiometric GECIs are those based on FRET. Among FRET-based GECIs, Cameleon probes have been widely used to monitor Ca^2+^ dynamics in the cytosol and subcellular compartments [16]. In Cameleon sensors, two FPs are linked together by a Ca^2+^-sensing peptide, designed based on calmodulin (CaM) and the CaM binding peptide of myosin light chain kinase M13 (M13) [17], separated by a glycyl-glycine linker. In this design, Ca^2+^ binding promotes the reversible association of CaM and M13, increasing energy transfer between the two FPs.

The initial Cameleon’s structure has been extensively modified in the following years in order to: (i) increase the brightness and dynamic range in response to Ca^2+^ changes; (ii) reduce the competing effect of endogenous CaM; (iii) obtain variants with different Ca^2+^ affinities [18,19]. In particular, in order to generate a probe suitable for the measurement of [Ca^2+^]_ER_, the binding interface between CaM and M13 was redesigned to decrease its affinity for Ca^2+^. This effort led to the development of a Ca^2+^ indicator (D1ER) with a relatively low Ca^2+^ affinity (K_d_s = 0.81 and 60 μM measured in vitro) that has been used to monitor [Ca^2+^] directly within the ER (or within the Sarcoplasmic reticulum, SR) of individual living cells [19] and live animals [20,21,22]. D1ER suffers, however, from several problems, in particular: (i) the K_d_ values calculated in vitro and in situ are substantially different; (ii) diverse in situ K_d_ values have been reported, depending on the cell model employed, i.e., 69 μM [23], 179 μM [24], and 200 μM [20]; (iii) D1ER has two K_d_s for Ca^2+^, one of high affinity, that practically reduces the effective dynamic range of the probe in the ER environment. Recently, a class of ER targeted “red-shifted” Cameleon probes have been generated [25]. Although their colour shift represents an important potential advancement in terms of simultaneous Ca^2+^ imaging of multiple organelles/parameters and low light-induced toxicity, presently available red-shifted Cameleons show a reduced dynamic range compared to the previously generated D1ER probe.

In 2006, the redesign of CaM/M13 interface led to the generation of a series of sensing domains (named D2, D3, and D4) with a wide range of Ca^2+^ affinities. One of this design, i.e., D4, when inserted in an Enhanced Cyan Fluorescent Protein (ECFP)/citrine-based Cameleon probe, show low affinity for Ca^2+^ in vitro. Importantly, D4 possesses a single K_d_ for Ca^2+^ (about 195 μM measured in vitro) [18].

A D4-based SR-targeted Ca^2+^ sensor has been generated by fusing the D4cpv probe to the SR resident protein calsequestrin, resulting in a Ca^2+^ sensor with several improvements compared to D1ER, such as a lower affinity for Ca^2+^ and a higher dynamic range [26]. The expression of the calsequestrin-D4 probe, however, causes a substantial increase in the luminal Ca^2+^ buffering capacity (about 50 Ca^2+^ binding sites per calsequestrin molecule) and the effect of this exogenous protein in the ER have not been investigated.

Recently, we and others reported the generation of two new D4-based ER-targeted Cameleon probes [5,27,28,29]. The sensor generated by Ravier and colleagues, named D4ER, was placed under the control of the rat insulin promoter, allowing specific β-cells confined expression. In this cellular environment, however, the authors reported several problems in the probe’s calibration: upon cell treatment with the Ca^2+^ ionophore ionomycin, or cell permeabilization by α-toxin or digitonin, the probe partially washed-out, making it impossible to determine accurate maximal and minimal fluorescence ratios. For this reason, all [Ca^2+^]_ER_ changes reported were expressed as emission fluorescence ratios (540/475) [27]. A similar ER-targeted sensor, named D4ER as well, was generated by our group and successfully used to measure [Ca^2+^]_ER_ changes in transfected mice cortical neurons [28,29]. In this case, the sensor was placed under the control of the ubiquitous cytomegalovirus (CMV) promoter, allowing its expression in different mammalian cell types. The same sensor has been also targeted to the ER lumen of plant cells by fusing the calreticulin 1A signal peptide of Arabidopsis upstream of the D4ER sequence, under the control of a single cauliflower mosaic virus (CaMV) 35S promoter. The resulting construct, CRT-D4ER, was transiently expressed in tobacco leaf epidermal cells and allowed the detection of induced decreases, as well as increases, in [Ca^2+^]_ER_ that were not reported by a CRT-D1ER probe, suggesting an improvement of the newly generated sensor in comparison to the previous one [5].

Here we report the characterization of the previously generated [28,29], ubiquitously expressible ER-targeted D4ER, the Cameleon probe based on the ECFP/citrine couple and the low affinity D4 Ca^2+^ sensing domain, with particular attention to its comparison with the pre-existing and widely used D1ER. The D4ER sensor has been characterized in situ in baby hamster kidney (BHK) cells, where its Ca^2+^ affinity and dynamic range has been determined. We also demonstrate that: (i) this ER probe is well capable of reporting [Ca^2+^] resting level in BHK and HeLa cell lines, whereas D1ER measurements are close to the probe’s saturating [Ca^2+^]; (ii) D4ER is able to report with much higher sensitivity (compared to its predecessor D1ER) changes in ER Ca^2+^ levels caused by the expression of a FAD-linked mutant form of PS2.

## 2. Materials and Methods

**Constructs:** D4ER generation is reported in [28]. Briefly, the Ca^2+^ sensing domain containing the mutant CaM and M13 of previously generated D1ER [19] was substituted with the low-affinity design D4. D4 was enzymatically cut from the D4cpv construct [18], using SphI/SacI enzymes.

Nuclear Ca^2+^ levels were monitored using the H2B-D3cpv probe, created by adding to the C-terminus of D3cpv, the human histone H2B gene targeting sequence. The latter was amplified from the cDNA encoding H2B-YFP (a kind gift of Dr. Teru Kanda, Gene Expression Laboratory, The Salk Institute for Biological Studies, La Jolla, CA, USA) by PCR, adding the restriction sites HindIII and NotI.

**Cell culture and transfection:** BHK and HeLa cells were grown in DMEM containing 10% FCS, supplemented with l-glutamine (2 mM), penicillin (100 U/mL), and streptomycin (100 µg/mL), in a humidified atmosphere containing 5% CO_2_. Cells were seeded onto glass coverslips (18 mm diameter) and transfection was performed at 60%–70% confluence using TransIT^®^-LT1 transfection reagent (Mirus Bio LCC, Madison, WI, USA) with 1 µg of total DNA (for co-transfections, 0.3 µg of Cameleon encoding plasmids plus 0.7 µg of pcDNA3 or PS2-T122R or mCherry-ER-3 (a gift from Michael Davidson, Addgene plasmid # 55041, Michael Davidson Lab The Florida State University Tallahassee, FL, USA)).

**Immunofluorescence and confocal microscopy analysis:** BHK cells grown on 18-mm coverslips at 50% confluence were transfected with D4ER encoding plasmid. After 24 h, cells were washed with PBS, fixed in 4% (*w*/*v*) formaldehyde for 10 min and incubated with 50 mM NH_4_Cl in Phosphate Buffered Saline (PBS). Cells were permeabilized for 4 min with 0.1% Triton X-100 in and blocked in blocking solution (PBS containing 2% (*w*/*v*) BSA, 10% goat serum, and 0.2% gelatin) for 1 h. Cells were incubated with the primary antibody (anti-Calreticulin; Pierce #PA3-900; 1:100, Thermo Fisher Scientific, Rockford, IL, USA) for 1 h at room temperature and washed three times with blocking solution. Alexa Fluor 555-conjugated secondary antibody (Invitrogen^TM^—Thermo Fisher Scientific, Rockford, IL, USA) was applied for 45 min at room temperature; then coverslips were washed three times with PBS and mounted using Mowiol 4–88 medium (Sigma-Aldrich, St. Louis, MO, USA). 

For co-localization analysis in live cells, coverslips were mounted into an open-topped chamber and maintained in an extracellular-like medium (see below). Images were collected on a Leica TCS-SP5-II confocal system (Leica Microsystems CMS GmbH, Wetzlar, Germany), equipped with a 100× oil objective (Plan Apo, NA 1.4, Leica Microsystems CMS GmbH, Wetzlar, Germany). For all images, pinhole was set to 1 Airy unit. The Argon laser line (488 nm) was used to excite D4ER FPs and the He/Ne 543 nm laser was used to excite either AlexaFluor 555-conjugated antibody or mCherry-ER-3. Confocal microscopy imaging was performed at 1024 × 1024 pixels per image, with a 200 Hz acquisition rate.

The co-localization index was expressed by Manders’ coefficient M1, calculated with the ImageJ co-localization analysis plug-in.

**Live cell imaging:** Cells expressing the fluorescent probes were analysed using a DM6000 inverted microscope (Leica Microsystems CMS GmbH, Wetzlar, Germany) with a 40× oil objective (HCX Plan Apo, NA 1.25, Leica Microsystems CMS GmbH, Wetzlar, Germany). Excitation light produced by a 410 nm LED (Led Engin #LZ1-00UA00 LED, LED Engin Inc., San jose, CA, USA ) was filtered at the appropriate wavelength (425 nm) through a band pass filter, and the emitted light was collected through a beamsplitter (OES s.r.l., Padua, Italy; emission filters HQ 480/40 M (for ECFP) and HQ 535/30 M (for citrine-YFP) and a dichroic mirror 515 DCXR). The beamsplitter permits the collection of the two emitted wavelengths at the same time, thus preventing any artefact due to movement of the organelles. All filters and dichroics were from Chroma Technologies (Chroma Technology Corporation, Bellows Falls, VT, USA). Images were acquired using an IM 1.4C cool camera (Jenoptik Optical Systems, Jupiter, FL, USA) attached to a 12-bit frame grabber. Synchronization of the excitation source and cool camera was performed through a control unit run by a custom-made software package, Roboscope (developed by Catalin Ciubotaru at VIMM, Padua, Italy); this software was also used for image analysis. Exposure time and frequency of image capture varied from 200 to 400 ms and from 2 to 5 s, respectively, depending on the intensity of the fluorescent signal of the cells analysed and on the speed of fluorescence changes.

Cells were mounted into an open-topped chamber and maintained in an extracellular-like medium (containing in mM: 135 NaCl, 5 KCl, 1 MgCl_2_, 0.4 KH_2_PO_4_, 1 MgSO_4_, 20 Hepes, 11 glucose, pH 7.4, 37 °C).

Classical experiments started in 1 mM CaCl_2_; after perfusion with 600 µM Ethylene glycol-bis(β-aminoethyl ether)-*N*,*N*,*N*′,*N*′-tetraacetic acid tetrasodium salt (EGTA), cells were stimulated by perfusion of bradykinin (BK, 100 nM) for BHK cells or histamine (Hist, 100 µM) for HeLa cells and cyclopiazonic acid (CPA, 20 µM); finally, digitonin (20 µM) was applied to obtain plasma membrane permeabilization in Ca^2+^-free intracellular-like medium (containing in mM: 130 KCl, 10 NaCl, 1 MgCl_2_, 2 succinic acid, and 20 Hepes, pH 7.05, 37 °C) and then a saturating CaCl_2_ concentration (3–4 mM) was applied. To prevent gross morphological changes of the ER upon permeabilization, dextran (5%) has been added to the intracellular-like medium.

**Figure 1:**
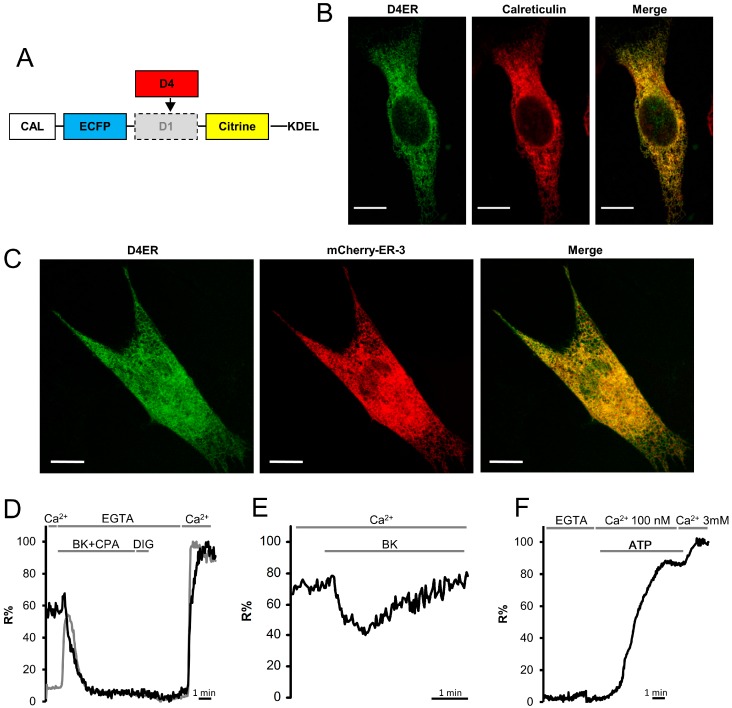
Generation and characterization of the ubiquitously-expressible ER-targeted probe D4ER. (**A**) Design of the construct codifying for D4ER; (**B**,**C**) D4ER correctly localizes in the ER; confocal images of BHK cells expressing: (**B**) the D4ER probe (green) and immuno-labelled with anti-calreticulin antibody (red); or (**C**) the D4ER probe (green) and an ER-targeted mCherry (red). Yellow colour represents the merge between the two signals coming from the protein pair. Scale bar, 10 µm; (**D**–**F**) D4ER correctly monitors [Ca^2+^] variations within the ER; (**D**) Representative kinetics of nuclear (grey) and ER (black) R% values in a single BHK cell co-expressing H2B-D3cpv and D4ER. Live cells were stimulated with BK (100 nM) plus CPA (20 μM) in a Ca^2+^-free extracellular-like medium, then permeabilized with digitonin (DIG, 20 µM) in an intracellular-like medium containing EGTA (600 µM); then an intracellular-like medium containing CaCl_2_ (3 mM) was perfused; (**E**) representative kinetics of ER R% values in a BHK cell expressing D4ER. Live cells were stimulated with BK (100 nM) in a Ca^2+^ (1 mM)-containing extracellular-like medium; and (**F**) representative D4ER kinetic of a BHK cell pre-incubated with CPA (20 μM) for 10 min in a extracellular-like Ca^2+^-free EGTA (300 μM)-containing medium, and then permeabilized for 30 s with DIG (20 μM) in an intracellular-like medium containing EGTA (600 μM). The cell was washed with an intracellular-like medium containing EGTA (600 μM) and perfused with an intracellular-like medium containing 100 nM free Ca^2+^ in the presence of ATP (100 μM). Eventually, Ca^2+^ in excess (3 mM) was added. Data are plotted as R%, as defined in the Materials and Methods section.

For Ca^2+^ pumping experiments (Figure 1F), a Ca^2+^-buffered solution was prepared by adding to the intracellular-like medium: *N*-(2-Hydroxyethyl)ethylenediamine-*N*,*N*′,*N*′-triacetic acid (H-EDTA), pyruvic acid, and MgCl_2_ (1 mM each), EGTA (2 mM), and CaCl_2_ (100 nM). ATP-Na (100 µM) was also added. The free [Ca^2+^] was estimated using the MaxChelator2.5 software and checked by fluorimetric measurements with Fura-2.

The reported ratio % values (hereafter named R%) represent 535/480-nm fluorescence emission intensities expressed as a % of the maximal ratio obtained in permeabilized cells upon perfusion of saturating CaCl_2_ concentration (see below). Values are plotted as a function of time or, for the probe calibration curve, of [Ca^2+^].

**Materials:** Restriction and modification enzymes were purchased from NEB (New England Biolabs, Inc. Ipswich, MA, USA) or Thermo Scientific (Thermo Fisher Scientific, Rockford, IL, USA). CPA, digitonin, bradykinin, histamine, ATP-Na, and dextran were purchased from Sigma-Aldrich (Sigma-Aldrich, St. Louis, MO, USA). All other materials were analytical or of the highest available grade.

**Statistical analysis:** Off-line analysis of FRET experiments was performed with ImageJ software. Citrine and ECFP images were subtracted of background signals and distinctly analysed after selecting proper regions of interest (ROIs) on each cell; subsequently, the ratio between citrine and ECFP mean emission intensities at 535 and 480 nm (R = F_535_/F_480_), respectively, were calculated. Data are presented as R%, where R% = (R − R_min_)/(R_max_ − R_min_) × 100 and R_max_ and R_min_ are the R values in permeabilized cells at saturating [Ca^2+^] and in the absence of Ca^2+^, respectively. For D4ER, the [Ca^2+^] was finally calculated as reported in [16] using a K_d_ of 321 µM and n (Hill coefficient) = 1.01; for D1ER, [Ca^2+^] was calculated as reported in [16] using a K_d1_ of 0.5 µM and n_1_ = 1.08 and a K_d2_ of 296 µM and n_2_ = 1.35.

All the presented data are mean values of at least three independent transfections. Calibration curve fitting was performed using Origin 8 SR5 (OriginLab Corporation). Averages are expressed as mean ± s.e.m. (N = number of independent experiments; * = *p* < 0.05, unpaired Student’s *t* test). For conversion of R% in [Ca^2+^], N represents the number of independent transfections.

## 3. Results and Discussion

### 3.1. Generation of D4ER

The ubiquitously-expressible, ER-targeted Cameleon probe was developed based on the previously generated D1ER [19], where the signal sequence from human calreticulin (MLLPVLLLGLLGAAAD) is fused upstream of the ECFP and the ER retention sequence (KDEL) is appended at the C-terminus of citrine. Although substitution of citrine with cpV has been shown to increase the dynamic range of a Cameleon probe by approximately five-fold [30], we decided to maintain citrine as the acceptor FP, since it has been demonstrated that, despite the presence of ER targeting and ER retention sequences, Cameleons containing cpV at the C-terminus show a poor ER localization [16,25]. The Ca^2+^ sensing domain containing CaM and M13 present in the D1ER probe (D1) was substituted with D4 [18], generating the D4ER probe (Figure 1A).

The fluorescence pattern of a typical BHK cell expressing D4ER is presented in Figure 1B. The distribution of fluorescence had the reticular pattern expected for ER and no diffuse cytoplasmic staining was observed. In order to confirm that D4ER has a selective ER localization, BHK cells were transiently transfected with the D4ER cDNA, fixed with formaldehyde, and then immuno-labelled with a *bona fide* ER marker (Calreticulin, CRT). The confocal images clearly show the D4ER signal perfectly overlaps with that of CRT labelling (Figure 1B). A quantitative analysis revealed a high average value of Manders’ colocalization coefficient, confirming a very good targeting of the probe to the ER compartment (M1 coefficient = 0.97 ± 0.01, mean ± s.e.m., N = 6). To exclude morphological artifacts caused by cell fixation that could influence probe localization, live BHK cells co-expressing the D4ER sensor, and an ER-targeted mCherry (mCherry-ER-3) were analysed for co-localization: also in this case, the distribution of the two fluorescent proteins perfectly overlaps (Figure 1C), indicating an excellent ER targeting of the probe (M1 coefficient = 0.95 ± 0.01, mean ± s.e.m., N = 12).

The functionality of the probe was then assessed in live BHK cells. Simultaneous recording of [Ca^2+^]_ER_ and nuclear [Ca^2+^] ([Ca^2+^]_n_) in single cells can be obtained by co-expressing the D4ER and a nucleus-targeted Cameleon (H2B-D3cpv, see Materials and Methods). Given that Ca^2+^ variations within the nucleus closely mirror those of the cytosol [31], the H2B-D3cpv probe can be used to indirectly evaluate Ca^2+^ changes in the cytosol ([Ca^2+^]_c_) in the very same cell co-expressing an organelle-targeted Ca^2+^ probe [32]. The IP_3_-generating agonist bradykinin (BK) was used as a stimulus in the presence of a SERCA inhibitor (cyclopiazonic acid, CPA), in order to elicit a complete release of Ca^2+^ from the ER (Figure 1D). The D4ER R% value (defined as described in Materials and Methods) decreased, while concomitantly that of H2B-D3cpv increased, indicating an effective ER Ca^2+^ mobilization that in turn results in a fast elevation of [Ca^2+^]_c_ (and thus of [Ca^2+^]_n_). Interestingly, the kinetics of the [Ca^2+^] changes in the two compartments were substantially different: the peak in nuclear signal occurred within 10–20 s, while the decrease in ER signal was maximal after 60–120 s. Thus, the maximal increase in cytosolic and nuclear [Ca^2+^] are reached when only a fraction (20%–30%) of the maximal decrease in ER signal has occurred. At the end of the experiment, permeabilized cells have been perfused with intracellular-like medium containing 600 µM EGTA, to get minimum R% value, and 3 mM CaCl_2_, to get the maximum R%.

The D4ER probe is also applicable to measure ER [Ca^2+^] variations in more physiological conditions, i.e., in cells bathed by a medium mimicking extracellular [Ca^2+^] (1 mM) and in the absence of SERCA inhibitors. The application of an IP3-generating stimulus (100 nM bradykinin, BK) induced a transient ER Ca^2+^ release, followed by the ER Ca^2+^ refilling (Figure 1E). The D4ER R% trace shows a decrease of about 40% upon BK stimulation, confirming the possibility to employ D4ER as a probe to investigate ER Ca^2+^ dynamics under physiological conditions.

The response of D4ER to [Ca^2+^]_ER_ changes was further evaluated in permeabilized cells (Figure 1F). BHK cells expressing D4ER were pre-treated with CPA to completely release Ca^2+^ from the ER and then permeabilized with digitonin in an intracellular-like medium containing no Ca^2+^ and 600 µM EGTA to get the minimum R% signal. CPA was washed away and the cells were then perfused with an intracellular-like medium with [Ca^2+^] buffered to 100 nM (to mimic [Ca^2+^]_c_ at rest) and ATP to fuel SERCA pumps. The D4ER signal started to increase as soon as exposed to ATP and a new steady state level, close to that observed in the intact live cell, was rapidly gained. Finally, 3 mM CaCl_2_ was perfused and this was followed by a further substantial R% increase. No further rise was observed upon perfusion of CaCl_2_ to 4 mM, while after about 60 s the signal started to decrease as the probe was lost from the cells (likely due to the toxicity of Ca^2+^ at such high level in permeabilized cells).

### 3.2. Calibration of D4ER: A Comparison with D1ER

**Figure 2:**
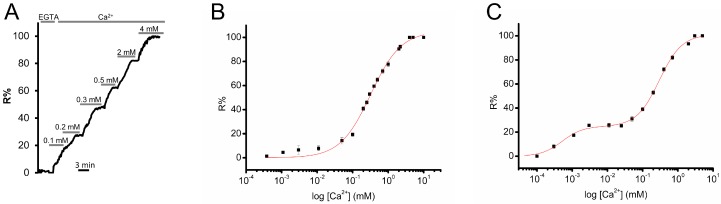
In situ calibration of the D4ER and D1ER probes. (**A**) Titration protocol. Representative kinetic of R% in permeabilized BHK cells transiently transfected with D4ER and exposed to different [Ca^2+^] in the medium. After ER emptying by BK (100 nM) and CPA (20 μM) application, cells were permeabilized with digitonin (20 μM) and bathed with an intracellular-like medium without energy sources and containing the indicated [Ca^2+^]; (**B**) in situ Ca^2+^ titration R% values measured with D4ER, along with corresponding fits of the data. Mean ± s.e.m, N ≥ 6 cells for each [Ca^2+^]; and (**C**) in situ Ca^2+^ titration R% values measured with D1ER, along with corresponding fits of the data. Mean ± s.e.m, N ≥ 6 cells for each [Ca^2+^].

The evaluation of the Ca^2+^ affinity (K_d_) provides an idea of the approximate range of Ca^2+^ concentrations in which a GECIs can effectively function as a Ca^2+^ indicator (reviewed in [33]). Moreover, it allows quantitative Ca^2+^ measurement, since it permits the conversion of R values in absolute [Ca^2+^] levels. To evaluate the Ca^2+^ affinity of D4ER, the calibration of the probe was performed in situ in permeabilized cells. BHK cells transfected with D4ER were permeabilized using digitonin (20 µM) in an intracellular-like medium containing no Ca^2+^, 600 µM EGTA, no ATP or other energy source. The permeabilized cells were then exposed to different [Ca^2+^], ranging from 0.4 μM to 5 mM, assuming that luminal [Ca^2+^] in the ER should, under these conditions, passively equilibrate with the Ca^2+^ of the perfused medium (Figure 2A). R% values were plotted against the log[Ca^2+^] applied. The calculated apparent K_d_ for D4ER is 321 μM (Figure 2B). The same protocol and analysis have been applied in order to evaluate D1ER Ca^2+^ affinity, since different in situ K_d_ values have been reported [20,23,24]. As showed before, D1ER has 2 K_d_s for Ca^2+^ of 0.5 μM and 296 μM, respectively (Figure 2C).

Altogether the data obtained show that D4ER displays a single K_d_ and lower affinity for Ca^2+^ compared to its predecessor D1ER, i.e., with a Ca^2+^ affinity more suitable for dynamically following Ca^2+^ variations within the organelle in live cells. To the best of our knowledge, the only two ER targeted GECIs available with a lower Ca^2+^ affinity, compared to D4ER, are the single fluorophore-based Ca^2+^ sensor CEPIA1er, with a reported K_d_ of 368 μM [11] and the very recently published GAP3-Aequorin, with a K_d_ of 489 ± 43 μM [15].

### 3.3. Dynamic Range and Resting ER Ca^2+^ Level Evaluation: Comparison between D1ER and D4ER

**Figure 3:**
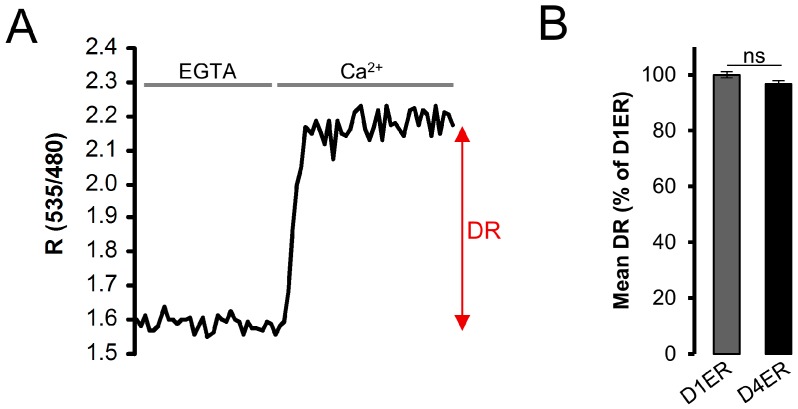
D1ER and D4ER dynamic range (DR) comparison. (**A**) BHK cells expressing D4ER were permeabilized with digitonin (20 μM) for 30 seconds in an intracellular-like medium containing EGTA (600 μM). Cells were then perfused with EGTA (600 μM), to record minimal R values and then with CaCl_2_ (3 mM), to get maximal values. A representative trace is presented as variations in ratio value between acceptor and donor fluorescence intensity (R); and (**B**) the histogram shows the DR, calculated as the ratio between R_max_ and R_min_, for D1ER and D4ER probes. Data are presented as mean ± s.e.m. (normalized to D1ER dynamic range); N ≥ 51 cells for each condition; ns (not statistically significant) = *p* > 0.1, unpaired Student’s *t* test.

One important parameter that must be evaluated both in the creation/modification of a Ca^2+^ indicator and in choosing the proper Ca^2+^ probe for specific measurements, is the probe’s dynamic range (DR). In ratiometric GECIs, the DR expresses the ratio between the maximal and the minimal R value obtainable with the probe and, thus, it is calculated as DR = R_max_/R_min_, where R_max_ is the 535/480-nm fluorescence emission ratio (acceptor/donor) obtained under Ca^2+^ saturated conditions, while R_min_ is the same ratio obtained in the absence of Ca^2+^ (Figure 3A; reviewed in [33]). To test whether the DR was changed by the substitution of D1 with D4, BHK cells were transfected either with D1ER or D4ER, permeabilized in intracellular-like Ca^2+^-free medium and then perfused with 600 μM EGTA, to get minimal R, or 3 mM CaCl_2_ to get maximal R values (Figure 3A). No significant difference was found between D1ER and D4ER dynamic range (Figure 3B). Noteworthy, however, is that part of the D1ER dynamic range (about 26%) is sensitive to Ca^2+^ changes from 0 to 10 µM, i.e., a range of concentration irrelevant for changes occurring physiologically in the organelle lumen.

Altogether, the above results show that the D4ER probe has a similar DR compared to D1ER, but a lower affinity for Ca^2+^. This represents an important improvement for a probe destined to measure ER Ca^2+^ concentration, which, at basal level, has been reported to reach, in some cell types, values even higher than 1 mM [34].

**Figure 4:**
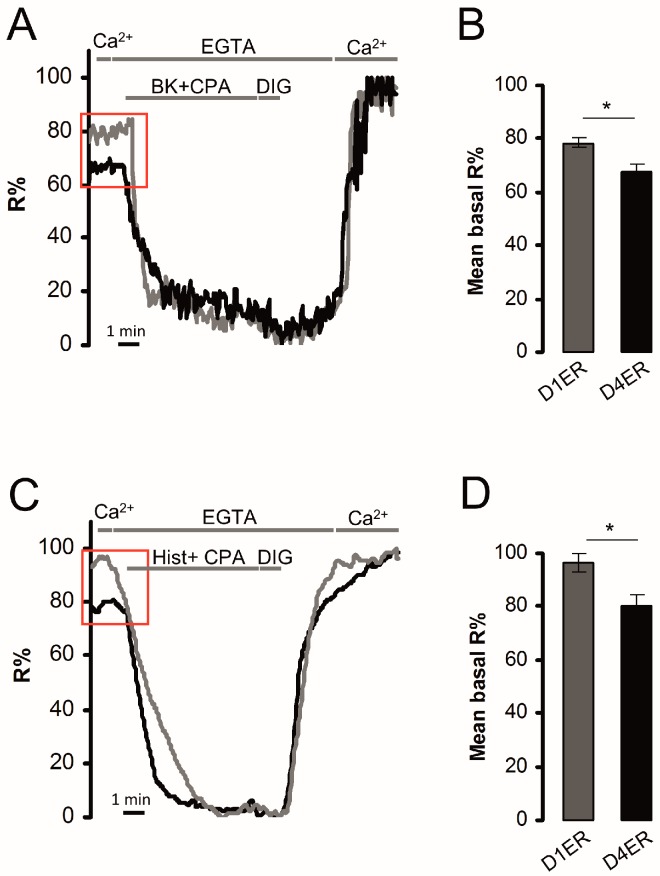
Basal ER Ca^2+^ level evaluation. (**A**) The protocol described in Figure 1D has been applied to BHK cells expressing either D1ER or D4ER. Representative traces of D1ER (grey) and D4ER (black) kinetics are presented as R%; (**B**) the histogram depicts basal mean ER R% values obtained with the two probes in BHK cells; mean ± s.e.m., N ≥ 38 cells for each condition; (**C**) representative kinetics of ER R% values in HeLa cells expressing D1ER (grey) or D4ER (black). Live cells were stimulated with Hist (100 µM) and CPA (20 μM) in a Ca^2+^-free extracellular-like medium. Cells were then permeabilized with DIG (20 µM) in an intracellular-like medium containing EGTA (600 µM); then, an intracellular-like medium containing CaCl_2_ (3 mM) was perfused; and (**D**) the histogram depicts the basal mean ER R% values obtained with the two probes in HeLa cells; mean ± s.e.m., N ≥ 25 cells for each condition, * = *p* < 0.05, unpaired Student’s *t* test.

In order to evaluate the entity of this improvement, we evaluated resting ER Ca^2+^ level in cells expressing either D1ER or D4ER probes (Figure 4A), employing the same protocol described in Figure 1C. Resting R% reported by D4ER were 67.8% ± 2.4% of R_max_ (mean ± s.e.m., N = 64). In terms of [Ca^2+^], the measured mean R% value correspond to 697.8 ± 16.1 µM (mean ± s.e.m., N = 3 independent transfections). D1ER R% levels at rest were 80.6% ± 2.8% of R_max_ (mean ± s.e.m., N = 19) (Figure 4B). In other words, ER Ca^2+^ levels measured by D1ER are closer to the probe’s saturating [Ca^2+^], compared to D4ER, confirming the improved suitability of D4ER as an ER Ca^2+^ sensor. An estimation of [Ca^2+^] measured by D1ER was obtained using the K_d_ obtained in our in situ calibration, resulting in an ER resting [Ca^2+^] value of about 633.7 ± 67.7 µM (mean ± s.e.m., N = 3 independent transfections), a value not very different from that obtained with D4ER. However, the calculated D1ER value is subjected to larger errors because: (i) as shown in Figure 4, R% values at rest are near the D1ER saturation and, thus, a conversion in terms of [Ca^2+^] is subjected to larger variability; and (ii) D1 shows a biphasic K_d_, making the calculation of [Ca^2+^] based on R% values more difficult.

The suitability of D4ER as an ER Ca^2+^ probe was strengthened by measuring resting [Ca^2+^] level in cells with reported higher [Ca^2+^]_ER_ [34], such as HeLa cells (Figure 4C). Resting R% reported by D4ER was 79.9% ± 4.7% of R_max_ (mean ± s.e.m., N = 25) (Figure 4D). In terms of [Ca^2+^], the calculated mean R% value corresponds to 1097.1 ± 200.2 µM (mean ± s.e.m., N = 3 independent transfections). The result obtained fits with what previously reported [34] using a low affinity ER-targeted aequorin. R% levels at rest measured using D1ER were 96.5% ± 3.8% of R_max_ (mean ± s.e.m., N = 26) (Figure 4D). The calculated R% is very close to probe’s saturation, preventing the possibility of calculating a reliable [Ca^2+^]. Altogether, the data obtained confirmed the improved performance of D4ER, compared to D1ER, as ER Ca^2+^ sensor. The improvement is particularly relevant in cells with high [Ca^2+^]_ER_ levels.

### 3.4. ER Ca^2+^ Concentration in FAD-Linked PS2-T122R Expressing Cells

**Figure 5:**
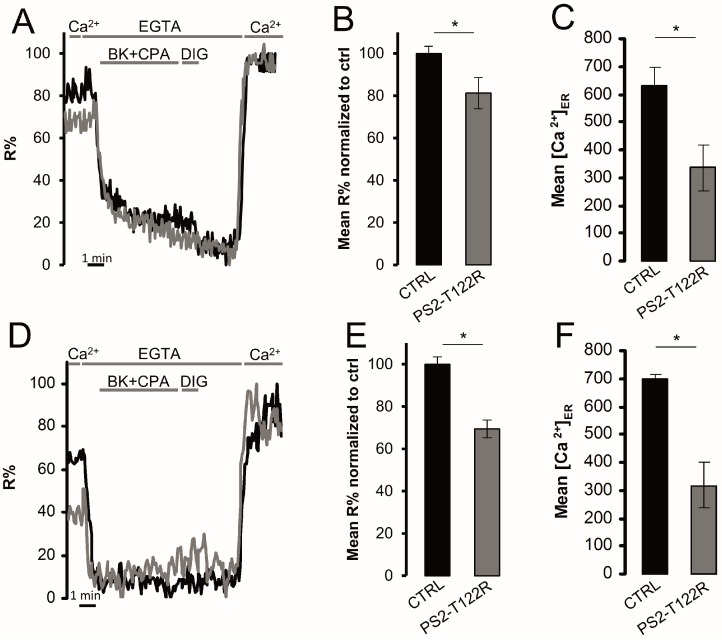
ER Ca^2+^ content variations upon PS2-T122R cell expression. The protocol described in Figure 1D has been applied to BHK cells co-expressing either D1ER (**A**–**C**) or D4ER (**D**–**F**) and PS2-T122R (grey traces) or the void vector (black traces), as controls; (**A**,**D**) representative traces are shown and represent R% variations upon different additions; (**B**,**E**) histograms show basal mean R% normalized to those obtained in void vector-transfected controls, in BHK cells expressing the D1ER (**B**) or D4ER (**E**) probe (mean ± s.e.m., N ≥ 16 cells for each condition); and (**C**,**F**) the conversion of calculated R% values in [Ca^2+^], measured using the D1ER (**C**) or D4ER (**F**) probe (mean ± s.e.m.; N ≥ 33). * = *p* < 0.05, unpaired Student’s *t* test.

The effect of FAD-linked PS2 mutations on ER [Ca^2+^] has been previously reported. In particular, by using aequorin-based Ca^2+^ probes in cell population measurements, we have previously shown that different FAD-PS2 mutants reduce ER and GA Ca^2+^ levels, mainly by inhibiting SERCA activity [35,36]. To verify the D4ER ability to trustworthy measure ER [Ca^2+^] variations, BHK cells expressing one of the most effective PS2 mutant (PS2-T122R [37]), or transfected with the void vector as a control, were evaluated for their ER Ca^2+^ content either by the D4ER or D1ER probe (Figure 5). After 1 min of recording of ER basal signal, release of Ca^2+^ from the organelle was elicited by applying the IP_3_-generating agonist BK in the presence of the SERCA inhibitor CPA; then, cells were permeabilized in an intracellular-like medium containing the Ca^2+^ chelator EGTA (600 µM), to obtain minimum R values. Maximum R was obtained by the further addition of CaCl_2_ (3mM; Figure 5A,D, for D1ER and D4ER, respectively). As expected, the expression of PS2-T122R strongly reduced ER Ca^2+^ level, compared to control cells, as revealed by both probes. Notably, while a reduction of 18.8%, in terms of R%, is observed in cells co-expressing PS2-T122R and D1ER, compared to controls (Figure 5B), this reduction is amplified using D4ER as a probe, causing a decrease of R%, compared to controls, of 30.6% (Figure 5E). In terms of [Ca^2+^], these latter variations represent a reduction from 633.7 ± 67.7 µM (mean ± s.e.m., N = 3 independent transfections ) or 697.8 ± 16.1 µM (mean ± s.e.m., N = 3 independent transfections) measured in control cells by D1ER or D4ER probe, respectively, to 336.0 ± 81.3 µM (mean ± s.e.m., N = 3 independent transfections) or 318.1 ± 82.1 µM (mean ± s.e.m., N = 3 independent transfections) measured in PS2-T122R expressing cells, by D1ER or D4ER probe, respectively (Figure 5C,F).

## 4. Conclusions

In this study, we characterized the recently generated, ubiquitously-expressible, ER-targeted D4ER Cameleon. This probe displays similar DR to that shown by the previously generated D1ER, but lower affinity for Ca^2+^ and, thus, it appears less prone to saturation in high Ca^2+^-containing organelles. Moreover, D4ER is characterized by a single K_d_ compared to the double K_d_ reported for D1ER. The latter aspect simplifies quantitative Ca^2+^ evaluation in intact live cells. Using the D4ER probe, the specific effect on intracellular Ca^2+^ stores due to the expression of the FAD-linked PS-T122R mutant was also evaluated. The data presented confirm previous results from our laboratory, showing that expression of FAD-PS2 reduces ER Ca^2+^ levels [30,31] and highlight the better suitability of the D4ER probe, compared to the pre-existing D1ER, for detecting differences in ER [Ca^2+^].
